# A case report of a secondary Takotsubo syndrome after sudden cardiac arrest in a teenager with LQTS2

**DOI:** 10.1093/ehjcr/ytag395

**Published:** 2026-05-29

**Authors:** Miltiadis Georgiadis, Athanasios Ziakos, Lars Kamper, Patrick Haage, Melchior Seyfarth, Nadine Abanador-Kamper

**Affiliations:** Department of Cardiology, Helios University Hospital Wuppertal, University Witten/Herdecke, Arrenberger Str. 20, Wuppertal 42117, Germany; Center for Clinical Medicine, Witten/Herdecke University Faculty of Health, Alfred-Herrhausen-Strasse 50, Witten 58455, Germany; Department of Cardiology, Helios University Hospital Wuppertal, University Witten/Herdecke, Arrenberger Str. 20, Wuppertal 42117, Germany; Center for Clinical Medicine, Witten/Herdecke University Faculty of Health, Alfred-Herrhausen-Strasse 50, Witten 58455, Germany; University Medical Center for Radiology, Helios University Hospital Wuppertal, University Witten/Herdecke, Heusnerstrasse 40, Wuppertal 42283, Germany; University Medical Center for Radiology, Helios University Hospital Wuppertal, University Witten/Herdecke, Heusnerstrasse 40, Wuppertal 42283, Germany; Department of Cardiology, Helios University Hospital Wuppertal, University Witten/Herdecke, Arrenberger Str. 20, Wuppertal 42117, Germany; Center for Clinical Medicine, Witten/Herdecke University Faculty of Health, Alfred-Herrhausen-Strasse 50, Witten 58455, Germany; Department of Cardiology, Helios University Hospital Wuppertal, University Witten/Herdecke, Arrenberger Str. 20, Wuppertal 42117, Germany; Center for Clinical Medicine, Witten/Herdecke University Faculty of Health, Alfred-Herrhausen-Strasse 50, Witten 58455, Germany

**Keywords:** Case report, Takotsubo syndrome, *KCNH2* gene, LQT-syndrome type 2, Extracorporeal life support, Cardiovascular magnetic resonance

## Abstract

**Background:**

Takotsubo syndrome (TTS) is a condition first identified in the 1990s in the Japanese population. It is believed that 1%–2% of acute coronary syndromes are due to TTS. The pathophysiological mechanism involves acute activation of the sympathetic nervous system with a cataclysmic release of catecholamines, causing acute myocardial dysfunction.

**Case summary:**

We report a case of an 18-year-old female patient who suffered an out-of-hospital cardiac arrest due to ventricular fibrillation of unknown etiology. Emergency cardiac catheterization revealed normal coronaries and hypokinesia of the apical segments. The patient was admitted to the ICU with refractory cardiogenic shock, necessitating extracorporeal life support measures. Serial echocardiograms showed rapid deterioration of left ventricular function. The diagnosis of TTS was confirmed with an early cardiovascular magnetic resonance study. A corrected QT interval (QTc) prolongation was observed intermittently. The patient showed an excellent neurological outcome and was discharged after implantable cardioverter defibrillator implantation for ambulatory care. Genetic testing revealed a previously unpublished *KCNH2* gene mutation. Mutations in this gene are known to cause long QT syndrome (LQTS).

**Discussion:**

Our case presents a secondary TTS after surviving a sudden cardiac arrest in a teenager with so far unknown LQTS2-associated ventricular fibrillation. We emphasize the importance of repeated electrocardiogram recordings and serial echocardiography in unexplained cardiogenic shock. Our case highlights the importance of an early multimodal therapeutic approach in the management of TTS to improve patient outcomes in this complex clinical entity.

Learning points

- Takotsubo syndrome can mimic acute coronary syndrome and should be considered as a cause of cardiogenic shock, even in younger patients with sudden deterioration.
- Long QT syndrome shows intermittent QT prolongation, predisposing to malignant arrhythmias; serial electrocardiograms and genetic testing are crucial for diagnosis, risk stratification, and family counseling.

## Introduction

Takotsubo syndrome is an acute but reversible cardiac syndrome characterized by acute heart failure symptoms that mimic the presentation of cardiac ischemia. The syndrome can occur after emotional or physical stress, and a secondary presentation is possible in the context of other ongoing pathologies. Takotsubo syndrome predominantly affects postmenopausal women; an analysis of the InterTAK Registry showed that more than 90% of patients are over 50 years of age at presentation.^1^ Presentation at a young age is extremely rare but can be complicated by serious adverse events.

The pathophysiology of the syndrome is not entirely clear, although evidence supports the involvement of stress-related hormone release leading to myocardial stunning.^2^ The typical imaging presentation, with apical ballooning, appears to result from regional variability in catecholamine receptor distribution across different ventricular segments.

Cardiac magnetic resonance (CMR) has been established as an important diagnostic modality for patients presenting with acute coronary syndrome and unobstructed coronary arteries, as is often the case in Takotsubo syndrome.^3^ A detailed review of the diagnostic workup of the syndrome and the InterTAK diagnostic score was proposed by an international expert committee of the ESC in 2018.^4^

Data from randomized trials on this condition remain scarce. This case report describes a rare instance of Takotsubo syndrome in a female teenager following cardiac arrest due to an arrhythmogenic syndrome.

## Summary figure

**Figure:**
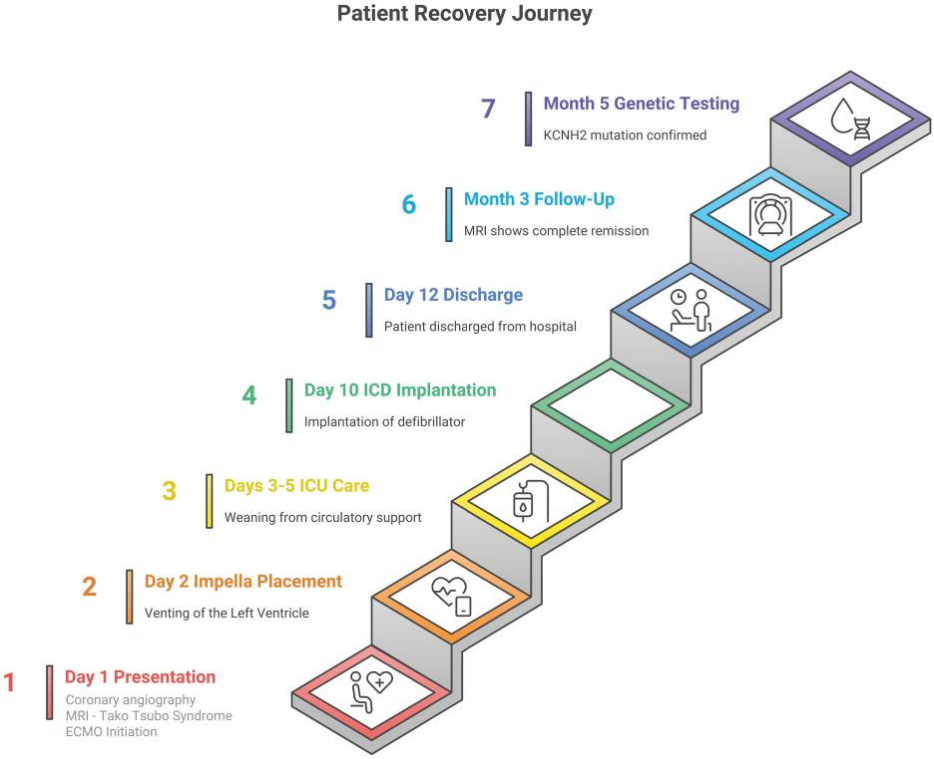


## Case presentation

**Figure 1:**
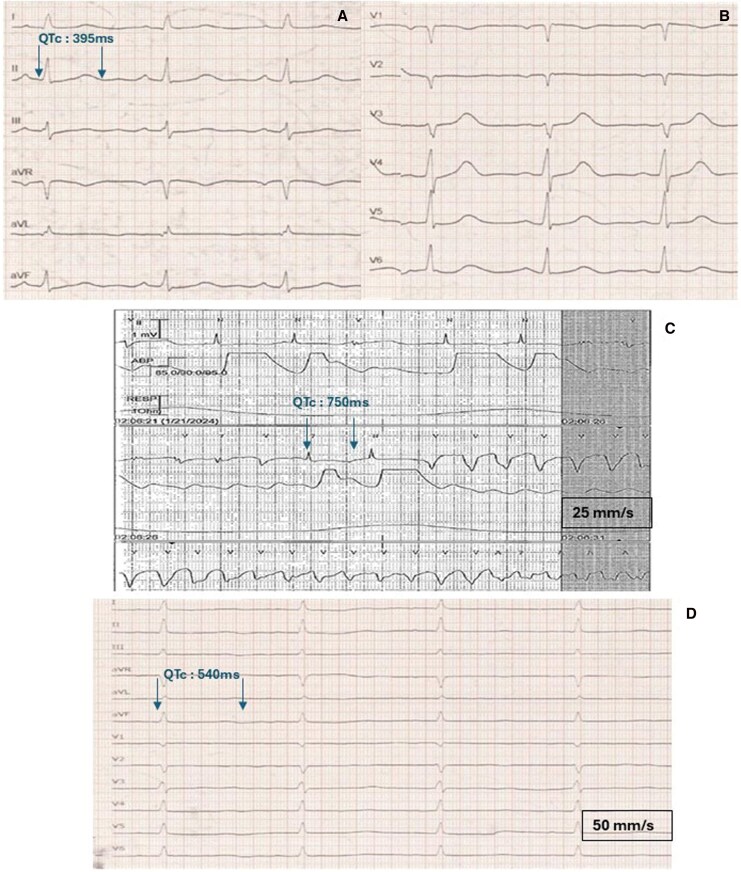
(A and B) Admission electrocardiogram showing a normal QTc. (C) Electrocardiogram monitoring in the intensive care unit demonstrating a prolonged QTc and progression into ventricular tachycardia. (D) Electrocardiogram recorder later during hospitalization, showing a prolonged QTc interval of 544 ms.

An 18-year-old female patient with no known past medical history presented to the emergency department after successful resuscitation by emergency services due to cardiac arrest with initial ventricular fibrillation. The electrocardiogram (ECG) obtained in the emergency department showed a normal QTc interval (*Figure 1*). Initial echocardiography revealed a mildly reduced left ventricular ejection fraction. Laboratory tests showed elevated cardiac troponin and creatine kinase levels, whereas electrolytes were within normal ranges. Intravenous magnesium administration had already been initiated by emergency medical services during transport to the hospital.

Early coronary angiography showed non-obstructive coronary arteries, while left ventriculography suggested hypokinesis of the inferior apical and inferolateral apical segments. Following coronary angiography, the patient was admitted to the ICU, due to progressively deteriorating hemodynamic parameters requiring advanced hemodynamic monitoring and the potential need for mechanical circulatory support. Serial echocardiographic examinations showed a rapid deterioration of left ventricular function, particularly involving the mid and apical segments.

**Figure 2:**
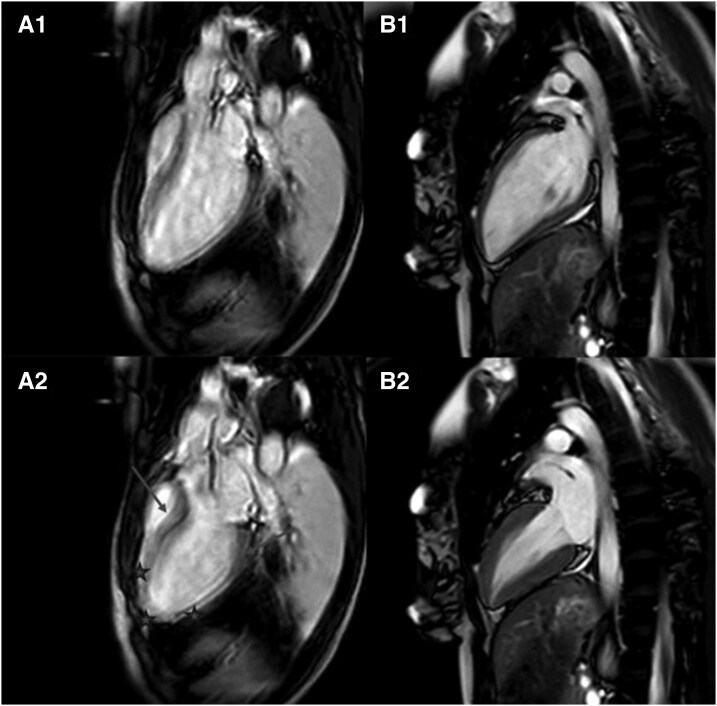
Cardiovascular magnetic resonance images demonstrate the apical ballooning in the acute phase (A1, A2), whereas in the control examination (B1, B2) a restoration of left ventricular function and ventricle geometry can be observed.

An urgent cardiac magnetic resonance (CMR) was performed, showing no late gadolinium enhancement, effectively ruling out ischemic injury due to thrombus passage or spontaneous coronary artery dissection. Dilated cardiomyopathy was considered in the differential diagnosis; however, the presence of myocardial edema in a pattern typical for Takotsubo syndrome, together with characteristic apical ballooning, strengthened the working diagnosis of Takotsubo syndrome (*Figure 2*).

A careful history obtained from family members and later from the patient herself revealed no emotional stressor at the time of the event. The patient had been quietly studying prior to her collapse, with no physical exertion or agitation that day and no prodromal symptoms such as chest pain or palpitations. She denied illicit drug use or recent medication intake. A whole-body CT scan excluded intracranial hemorrhage, aortic dissection, and pulmonary embolism. Toxicology screening was negative. In the absence of identifiable triggers, the presentation was attributed to Takotsubo syndrome occurring in the context of out-of-hospital cardiac arrest and ventricular fibrillation.

During her ICU course, the patient experienced recurrent episodes of polymorphic ventricular tachycardia. Although her initial post-arrest ECG showed a normal QTc interval, intermittent QTc prolongation—reaching up to 750 ms—was observed on some recordings (Figure 1). No acquired cause of QTc prolongation could be identified.

Because of refractory cardiogenic shock, venoarterial extracorporeal membrane oxygenation (VA-ECMO) was initiated approximately 5 h after admission. This stabilized the patient’s hemodynamics; however, persistent severe left ventricular dilatation necessitated venting of the left ventricle, and an Impella CP® device was placed the following day. Over the next 5 days, the patient’s hemodynamic parameters improved, enabling successful weaning and removal of the support systems. Following hemodynamic stabilization, beta-blocker therapy was initiated in the patient in accordance with current recommendations for the management of long QT syndrome (LQTS). The patient demonstrated an excellent neurological outcome.

**Figure 3:**
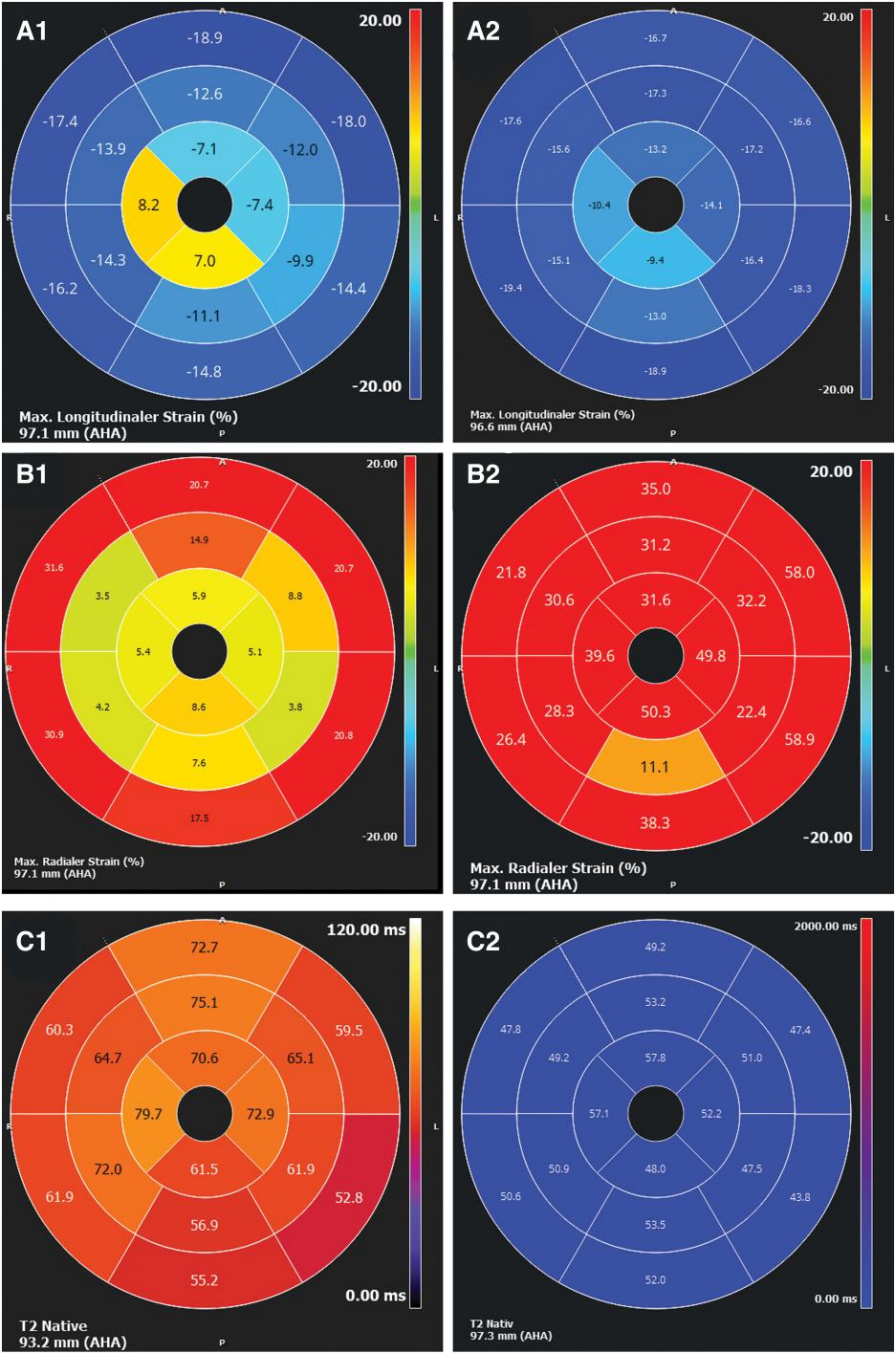
A1/A2 and B1/B2: polar maps showing the regional distribution of myocardial dysfunction using myocardial strain analysis (feature tracking) in the acute phase (A1/B1) and in the follow-up examination (A2/B2). C1/C2: myocardial edema imaging with T2-mapping (GraSE) in the acute phase (C1) and return to normal values in the control examination.

A follow-up CMR 1 week after the event showed normalization of left ventricular ejection fraction and resolution of ventricular dilatation. T1 and T2 mapping values had decreased globally compared with the acute phase but had not completely normalized. These findings were consistent with Takotsubo syndrome. No structural abnormalities suggestive of arrhythmogenic cardiomyopathy were detected (*Figures 2* and *3*, *Table 1*).

**Table 1:** Volumes and function

	Acute phase	Follow-up
LVEDVI	105 mL/m^2^	76 mL
LVEDV	180 mL	126 mL
LV-ESV	116 mL	40 mL
LV-SV	59 mL	86 mL
LV-EF	34%	68%
GLS/GCS/GRS	−13, 3%/−6, 1%/8, 1%	−20, 1%/19, 2%/32, 6%

CMR volumetric and functional values at the time of the incident in comparison with metrics 3 months later showing the transient nature of the changes, highlighting the return of myocardial function and normal ventricle geometry.

CMR, cardiovascular magnetic resonance; LVEDVI, left ventricular end-diastolic volume index; LVEDV, left ventricular end-diastolic volume; LV-ESV, left ventricular end-systolic volume; LV-SV, left ventricular stroke volume; LV-EF, left ventricular ejection fraction; GLS, global longitudinal strain; GCS, global circumferential strain; GRS, global radial strain.

Prior to discharge, an implantable cardioverter defibrillator (ICD) was implanted for secondary prevention given the presentation with ventricular fibrillation cardiac arrest in the setting of suspected inherited arrhythmia syndrome.

Genetic testing revealed a previously unpublished *KCNH2* gene mutation (c.1785del, p.(Lys595Asnfs*18)). This mutation results in a frameshift and protein truncation of a critical potassium channel subunit involved in cardiac repolarization. Given the functional importance of this region, the variant is classified as having a high likelihood of pathogenicity and is consistent with a long QT syndrome type 2 (LQTS2) phenotype. The mutation was not found in PubMed, HGMD, ClinVar, or gnomAD databases. Cascade family screening identified the same *KCNH2* mutation in the patient’s father and 2 brothers. They underwent cardiological evaluation including ECG, echocardiography, repeated 24-h Holter monitoring, and exercise testing. Although no clinically significant abnormalities were identified, they were classified as genotype-positive individuals carrying the same mutation. In accordance with current ESC guideline recommendations for the management of LQTS, they were referred for genetic counseling and longitudinal cardiological follow-up, and beta-blocker therapy was initiated despite the absence of overt QT prolongation.

## Discussion

This case represents secondary Takotsubo syndrome triggered by an extreme physiological stressor, namely sudden cardiac arrest. The patient’s pronounced QTc prolongation, which most likely precipitated ventricular fibrillation and cardiac arrest, was intermittent and not evident on the initial ECG, highlighting the importance of serial ECG recordings and a high index of suspicion for latent QT prolongation. Genotype-confirmed patients with concealed LQTS account for approximately 25% of the at-risk LQTS population.^5^ Accordingly, all patients with suspected LQTS should undergo evaluation at specialized centers with genetic testing.^6^

*KCNH2* encodes the rapid delayed rectifier potassium channel (hERG/IKr), which plays a central role in cardiac repolarization. Pathogenic variants in this gene cause LQT2, typically inherited in an autosomal dominant manner, and predispose carriers to QT prolongation and polymorphic ventricular arrhythmias, albeit with variable penetrance and expression.^7^

Beyond classic channelopathy phenotypes, *KCNH2* mutations and QT prolongation have also been reported in patients with specific arrhythmogenic cardiomyopathy phenotypes, including dilated cardiomyopathy, left-dominant arrhythmogenic cardiomyopathy, and ventricular non-compaction.^8,9^ These observations suggest that KCNH2 variants may contribute to arrhythmogenic substrates beyond isolated repolarization abnormalities. The 2019 Heart Rhythm Society consensus statement on arrhythmogenic cardiomyopathy listed KCNH2 among genes potentially involved in these conditions.^10^ In our case, no imaging evidence of cardiomyopathy was identified. The variant detected may represent a novel, family-specific (‘private’) mutation, which can still exert pathogenic effects, particularly when resulting in protein truncation and loss of function. Similar patterns of private mutations are well described in inherited cardiac diseases such as arrhythmogenic right ventricular cardiomyopathy.^11^

Clinically, this case highlights the need for vigilance in survivors of cardiac arrest with rapidly progressive ventricular dysfunction, in whom Takotsubo syndrome should be considered. Early mechanical circulatory support with careful adaptation to hemodynamic and loading conditions may be lifesaving in selected cases. However, dedicated studies in this population are lacking, and further research is needed to define optimal selection criteria and management strategies.

In conclusion, this case describes a rare presentation of Takotsubo syndrome in a teenager following ventricular fibrillation cardiac arrest in the setting of an underlying KCNH2 mutation. The intermittent nature of QTc prolongation underscores the diagnostic challenge of latent LQTS and the importance of repeated ECG monitoring. The coexistence of a pathogenic channelopathy and severe stress-induced myocardial dysfunction highlights a complex interaction between arrhythmogenic susceptibility and acute adrenergic stress, emphasizing the value of comprehensive phenotypic assessment, genetic evaluation, and family screening in young patients with unexplained cardiac arrest.

## Supplementary Material

ytag395_Supplementary_Data

## Data Availability

All data acquired during the study are available in anonymized form upon reasonable request from the corresponding author.

## Lead author biography

Mr Georgiadis is a board-certified cardiologist trained at Helios University Clinic Wuppertal, who has received training in medical genetics and has conducted research in cardiac magnetic resonance imaging. His primary interests include cardiovascular imaging, medical genetics, cardiomyopathies, and structural heart disease.

## Notes

**Consent:** Written informed consent for publication of this case report, including clinical details and any accompanying images, was obtained from the patient. This report complies with the ethical standards outlined by the Committee on Publication Ethics (COPE).
